# The added value of implicit motives for management research Development and first validation of a Brief Implicit Association Test (BIAT) for the measurement of implicit motives

**DOI:** 10.1371/journal.pone.0198094

**Published:** 2018-06-20

**Authors:** Hendrik Slabbinck, Arjen van Witteloostuijn, Julie Hermans, Johanna Vanderstraeten, Marcus Dejardin, Jacqueline Brassey, Dendi Ramdani

**Affiliations:** 1 Department of Marketing, Ghent University, Gent, Belgium; 2 School of Business and Economics, VU Amsterdam, Amsterdam, the Netherlands; 3 Antwerp Management School, Antwerp, Belgium; 4 Department of Management, University of Antwerp, Antwerp, Belgium; 5 CERPE, Department of Economics, University of Namur, Namur, Belgium; 6 Faculty of Economic, Social and Political Sciences and Communication (ESPO), Université catholique de Louvain, Louvain-la-Neuve, Belgium; 7 Louvain Research Institute in Management and Organizations (LouRIM), Université catholique de Louvain, Louvain-la-Neuve, Belgium; Eberhard-Karls-Universitat Tubingen Medizinische Fakultat, GERMANY

## Abstract

Many Management (sub-)disciplines, from Organizational Behavior and Marketing to Accounting and Strategy, are interested in antecedents and consequences of individual attitudes and traits. A key aspect of personality profiles are explicit and implicit motives. Yet, Management scholars mainly focus on explicit motives, with limited attention to implicit motives. We argue that this state of affairs probably came into being because current Management researchers mainly rely on implicit motive measures that are either difficult to apply or to develop, hampering researchers from applying implicit motive measures. To overcome the downsides of available instruments, we develop a Brief Implicit Association Test (BIAT) as an efficient, reliable and valid measure of implicit motives, particularly the needs for achievement, affiliation and power. To explore our BIAT’s predictive validity, we apply this measure to a specific research domain within Management: Entrepreneurship. We examine implicit motives’ association with entrepreneurial self-efficacy, business founding, and financial profitability. Our results show that the introduction of implicit motives can unlock stranded discussions in this research domain. Overall, we argue that implicit motives can help to push the boundaries of the study of deep-level attributes in a wide range of organizational and managerial settings.

## Introduction

Identifying and quantifying the motives of decision-makers is a key aspect in many disciplines of Management research, varying from work floor employees in Organizational Behavior to upper echelon managers in Strategy [1–4]. Although Organizational Behavior researchers have acknowledged that motives may also have an unconscious, implicit aspect [2,3,5,6], many if not most researchers in Management research only assess explicit motives while their implicit counterparts remain largely untapped. This implies a missed opportunity because an impressive body of research in Psychology and a slow but steadily growing stream of research in Management clearly show that these implicit motives influence many business, economic, political and societal phenomena independent and different from (or in interaction with) motivational dispositions that people attribute explicitly to themselves at a conscious level [7–11]. A plausible reason for this lack of Management research into implicit motives is that measurement instruments that are relatively easy to develop and/or administer were missing until recently.

The main goal of this paper is to offer a good solution that may boost implicit motives research in the Management domain. Towards this end, we first discuss the concept of implicit motives, arguing why they are different from their explicit counterparts, and provide a short overview of the traditional and new methods for the assessment of implicit motives, including their applicability in Management research. We then show how recent developments in (implicit) motives measurement can stimulate and encourage scholars in Management’s many sub-disciplines to adopt measures of implicit motives in field settings. Subsequently, we introduce the Brief Implicit Association Test (BIAT) [12] and provide a first validation of the BIAT as a measure of implicit motives, particularly the needs for achievement, affiliation and power. We test its predictive validity in the context of a study into entrepreneurial self-efficacy, business founding, and financial profitability.

Our key argument is that the BIAT provides an instrument that is highly complementary to both explicit measures and other implicit measures that are currently used in Management research, such as the Conditional Reasoning Test (CRT) or the Implicit-PsyCap Questionnaire (I-PCQ) [2,13]. The CRT and I-PCQ are other promising implicit measures that were specifically developed for the use in Management research [13]. Compared to the CRT, however, the BIAT is very flexible, and very easy to develop, offering ample opportunities to design new versions probing into a wide class of different motives. Moreover, contrary to the I-PCQ methodology, the psychometric properties of the BIAT is much better validated. The I-PCQ and CRT also belong to a different class of implicit measures than the BIAT. As different classes of implicit motives may predict different types of behavior, the introduction of the BIAT into Management research will most likely yield new insights in management theory and practices [2,3,5,6].We return to these issues, and many others, in much greater detail below.

Stimulating implicit motives in Management research is very promising indeed, given the modern psychological insights into the differential effects of explicit versus implicit motives on attitudes, behaviors and outcomes. By way of illustration, we argue that research has failed to provide compelling empirical support for the argument that an entrepreneur’s motives, particularly her or his (explicit) need for achievement, can explain key aspects of entrepreneurship [14–16]. We argue and show that this is different when both explicit and implicit motives are included. In the Conclusion and Discussion, we elaborate on a few limitations of our research, and highlight potential directions for future work within the broad Management domain.

### Implicit motives and their assessment

Current Personality Psychology distinguishes between two major elements of personality that affect and shape mental processes and behavior, namely personality characteristics or *traits* and motivational dispositions or *motives* [17,18]. These two distinct aspects of personality–traits and motives–have led separate lives, each developing its own measurement instruments. Yet, both streams of research converge on the idea that behavior is energized and directed by two distinct types of personality systems–namely an implicit system that operates outside of conscious awareness and control, and an explicit system that functions at a conscious level [19,20]. Indeed, implicit and explicit motives differ in their impact on behavior. In general, implicit motives predict spontaneous, uncontrolled behavior, and effort-related task performance, whereas explicit motives predict behavior that is subject to conscious thought and deliberation, such as self-reflective appraisals, judgments and deliberate choices [19,21]. For example, Brunstein and Schmitt [22] showed that the implicit motive to achieve was independent from the explicit motive to achieve, and that each type of motive predicted different types of behavior. That is, the implicit achievement motive exclusively predicted how hard participants tried to improve their performance on a task (i.e., effort-related task performance), whereas the explicit achievement motive uniquely predicted how much participants liked the task (i.e., self-reflective appraisal).

On the one hand, given the conscious representation of the explicit system, explicit motives can be easily assessed via self-reports, by use of questionnaires. These measures of explicit motives are well-established in the Management research domain, including Entrepreneurship [23]. On the other hand, however, because implicit motives operate outside of a person’s awareness and people often lack direct introspective insight into their implicit system, sensitive indirect measurement instruments are needed to measure these hidden individual differences [24,25]. Indeed, over the course of time, several measurement procedures have been proposed for the assessment of implicit motives. However, for reasons we will discuss below, the implicit motives measurement instruments are rarely applied in Management research [2,3], including Entrepreneurship [26].

The assessment of implicit motives can be traced back to the 1930s with the development of the Thematic Apperception Test (TAT) [27]. The TAT was developed to explore the unconscious dynamics of personality. Murray believed that people share the same basic sets of 27 motives, and differ only in their priority ranking of these needs [27,28]. He further argued that motives cannot be observed directly, but instead must be inferred indirectly. For this purpose, he developed the TAT. The TAT is a projective test that presents research participants with a series of ambiguous pictures. The participant is asked to spontaneously develop a story for each picture. The assumption is that the participant projects her or his own needs into the story, so that the stories can be analyzed and interpreted to uncover each participant’s underlying needs. For example, Atkinson and McClelland [29] find that the frequency of food-related interpretations of TAT pictures relates positively to the time that had passed since participants had their last meal.

A criticism of this early work has been that the classification of motives is just a lengthy inventory of needs, lacking sufficient empirical or theoretical evidence [28]. Because of their relevance for understanding economical and societal life, implicit motives researchers focused on Murray’s power, affiliation, and achievement needs [9]. Even though other basic motivational needs, such as hunger or sex, may exist, one of the greatest achievements of the early implicit motives researchers is that they developed an empirically validated system of content analysis for motives imagery and transformed the TAT into a major tool for scientific personality psychology [9,30–32]. To distinguish the original TAT from the version used to assess implicit motives, researchers started to refer to the latter as the Picture Story Exercise (PSE) [33,34]. To avoid any misunderstanding, we use the term PSE to refer to the TAT version developed to assess implicit motives.

A typical PSE consists of four to six pictures that depict people in a variety of social settings. For each picture, participants write an imaginative story. The content of these imaginative stories can be coded according to motivational coding systems, empirically derived and refined over decades [35,36]. The summed scores yield the person’s overall implicit motives scores. Even though recent work confirms the good psychometric qualities of PSE measures and other content-coding and interpretivist techniques [33,34,37,38], many researchers remain highly skeptical toward these so-called ‘subjective’ techniques [39–41]. Next to the criticisms on the so-called subjective nature of these techniques, also the heavy administrative and time-consuming data preparation procedures hamper a wider application of these techniques [42]. To illustrate, to complete a default PSE, each participant has to write stories about four to eight pictures, and each picture presentation and story-writing episode is advised to last about four to five minutes [33]. Afterwards, stories have to be independently content-coded by two or more trained coders. An experienced coder needs between two and five minutes to score one PSE story. Thus, a typical PSE with 6 picture presentations and 60 participants requires up to 30 hours of data collection and 12–30 hours of data preparation, and this only to assess the participants’ implicit motivational dispositions [33].

The Operant Motive Test (OMT) which can be seen as a modified PSE technique [43] partially addresses the shortcomings of the PSE. In a typical OMT, participants review 4–15 pictures and invent a story (without having to write it down). Then they offer spontaneous associations, in response to the following questions: (1) “What is important for the person in this situation and what is the person doing?”; (2) “How does the person feel?”; (3) “Why does the person feel this way?”; and (4) “How does the story end?” Next to motives imagery, the OMT scores also reflect motives-relevant traits (i.e., an implementation strategy of a motive), which should increase their predictive validity and test-retest reliability for motives measures. Recent studies on the validity of the OMT confirm its good psychometric qualities [11,44–47]. Yet, compared to the PSE, the administration of the OMT is indeed faster and renders shorter answers.

However, the OMT still requires relatively much effort of research participants who need to write down multiple sentences. Moreover, akin to the PSE, the OMT may also provoke resistance and skepticism, leading to disengaged or defensive responding by employees, entrepreneurs, managers or other participants in Management research [23]. Consequently, when new, yet ‘objective’ indirect measures were developed in the 1990s, offering an alternative–cheaper, quicker, and shorter–method, many researchers abandoned the subjective content-coding and interpretivist techniques in favor of the new objective techniques [39]. Researchers in implicit motives adopted these techniques, too, and provided new theoretical insights in the mechanisms underlying motives, behaviors and outcomes.

By now, a large number of new implicit assessment techniques circulate in the literature. In Management research, rooted in industrial and organizational psychology, excellent reviews are offered by Bing, LeBreton, Davidson, Migetz, and James [39], and Uhlmann and colleagues [23]; in personality and social psychology, examples of insightful overviews are De Houwer and colleagues [48], and Gawronski and De Houwer [49]. Perhaps strikingly, both domains–industrial and organizational psychology, on the one hand, and personality and social psychology, on the other hand–are not really connected, as signaled by the lack of cross-references. For example, the most popular new implicit measure for the assessment of implicit motives that is developed by Organizational and Management Researchers is the Conditional Reasoning Test [39], whereas the most popular technique that stems from Psychological researchers is the Implicit Association Test (IAT) [50].Yet, the CRT technique is not frequently referred to in the personality and social psychology literature, whilst the IAT instrument is not widely applied in the industrial and organizational psychology literature.

In what follows, we will first discuss the IAT and CRT, along with two other promising implicit measures that can be used for the assessment of implicit motives: the Brief Implicit Association Test [12] and the Implicit PsyCap Questionnaire [2]. We then argue why Management research may benefit especially from the introduction of the BIAT.

#### Implicit Association Test (IAT) and Brief Implicit Association Test (BIAT)

The IAT is a computerized response latency task that is assumed to measure the relative strengths of associations amongst two pairs of contrasted concepts (e.g., ‘positive–negative’ and ‘sunshine–rain’). In IAT studies, stimuli that belong to one of the concepts are presented on a computer screen one by one. These stimuli have to be allocated to the concept to which they belong by pressing one of two response keys. Results show that responses are faster when associated categories are assigned to the same response key than when non-associated categories are assigned to the same response key.

For example, people with a positive attitude toward sunshine are faster in allocating stimuli when they have to press the same response key for stimuli that belong the ‘sunshine’ or ‘positive’ concept than when they have to press the same response key for stimuli that belong to the ‘sunshine’ or ‘negative’ concept. Assuming that attitudes are represented in memory as associations between target concepts (e.g., sunshine versus rain) and attribute concepts (e.g., positive versus negative), performance on the IAT task reflects someone’s evaluative associations that underlie implicit attitudes [50]. The IAT was initially set up for the assessment of implicit attitudes, but has been used to assess a multitude of other concepts since then, including personality traits and implicit motives [22,51–54].

The administration and data processing of the IAT take considerably less time than that of the PSE or OMT. Yet, in comparison with self-reported measures, they still require considerably more effort to gather data and to prepare data for analysis. For example, the administration of one implicit motive by means of the IAT easily takes between five and ten minutes, whereas self-reported measures of equivalent constructs only require a fraction of this time [12]. As an answer to this shortcoming, the Brief Implicit Association Test [12], or BIAT, was developed. The BIAT shortens the time required to measure implicit associations, while retaining the valuable psychometric properties of the IAT. The BIAT can be completed in little over a minute [55]. Bar-Anan et al. [56] concluded on the basis of eight evaluation criteria, across three topics and using a very large sample (n = 23,413), that the BIAT has a high test-retest reliability, is internally consistent, and is relatively well resistant to strategic and volitional influences. In addition, researchers who want to develop new BIAT versions can rely on a vast amount of IAT research, including ready-to-use research materials for the assessment of implicit motives, personality traits, stereotypes, attitudes, and societal and ethical dilemmas.

#### The Conditional Reasoning Test (CRT) and the I-PCQ

A Conditional Reasoning Test is an implicit measurement procedure that relies on the premise that individuals unconsciously apply justification mechanisms to rationalize behaviors and thoughts that deviate from social norms. These justification mechanisms cause people to frame norm-violating behaviors and thoughts in such a way that the motive to engage in such norm-violating behaviors and thoughts appears to be the most rational course of action. A CRT assesses these unconscious motives by presenting people with reasoning exercises with predefined solutions that seem equally logical. The presented solutions are based on justification mechanisms. Thus, the pattern of selected solutions is indicative of the justification mechanisms that people apply, and hence of the underlying unconscious or implicit processes through which people motivate their behaviors and thoughts. CRTs have been successfully developed and used for the assessment of achievement motivation [13], aggression [57], power [58], creativity [59], addiction proneness [60], and most likely also for a number of other constructs. Recent studies, including two meta-analyses, show that the CRT has good psychometric qualities [60–63]. Notwithstanding these good properties, the development and validation of new CRTs is rather effortful [39] and completion of one CRT requires up to 20–30 minutes [59].

The Implicit Psycological Capital Questionnaire (I-PSQ) may serve as a good alternative for the assessment of implicit motives. The I-PCQ is a semi-projective measure that relies on a short and easy-to-score methodology. That is, participants are presented short statements and requested to invent stories about the people in these statements. Each statement is followed by normal, short construct-targeted questions scored along a Likert scale. To date, the I-PCQ has been developed only for the assessment of PsyCap, but the same semi-projective methodology could easily be applied to alternative constructs such as implicit motives. Yet, such a to-be-developed I-PCQ must be validated sufficiently (e.g., experimental arousal effects; convergent validity with other implicit motive measures; similar range of predictive validity as other implicit motive measures; careful description and evaluation of test characteristics; et cetera) before I-PCQ type of measures can supplement or replace existing implicit motive measures.

In this paper, without discrediting the CRT, IPCQ or any other method, we argue in favor of the use of the BIAT in the context of Management research, with reference to three main arguments. IAT and related techniques are complementary from a theoretical perspective. That is, different types of implicit measures may activate different elements in a person’s implicit motives network. The BIAT can be classified as a non-symbolic measure, and the OMT, CRT and I-PCQ as symbolic measures [64]. Scores of non-symbolic measures are derived from the physical feature of the measurement procedure (i.e., the response latencies for the BIAT), whereas scores of symbolic measures are derived from the interpretation that participants give to stimuli (i.e., the way in which participants interpret the OMT pictures, CRT scenarios or I-PCQ questions). Studies on the relationships between different types of implicit measures are relatively scarce. Yet, all these studies have in common that the found correlations between different types of indirect measures are relatively low and that both types of measures predict unique portions of variation in outcome measures.

For example, Bosson, Swann, and Pennebaker [65] administered implicit self-esteem by means of symbolic and non-symbolic indirect measures, finding no correlations amongst both types of measures. Similarly, Bar-Anan and Nosek [66] compared the performance of seven indirect measures across several domains. Six of their measures, including the IAT and BIAT, were non-symbolic measures and only one was a symbolic measure (The Affect Misattribution Procedure, or AMP) [67]. The average correlation between the AMP and the other indirect measures only reached .26. Slabbinck and colleagues [54] provided parallel results. They measured the implicit need for power by means of the IAT and the PSE, reporting a correlation of .31 between both measures. They further showed that the IAT had incremental validity in predicting behavior over and above the PSE.

It is important to note that the low to mediocre correlations between both types of measures says little about the validity of both types of measures. A lack of relationship may indicate assessment of (partially) different constructs, and not lack of validity of either one or both [54,66]. A plausible and regularly suggested explanation as to why different types of indirect measures have little in common, is that each type of indirect measure relates to a different underlying process [19,54,64,65]. Several proposals have been put forward regarding the processes underlying the (B)IAT scores. The available evidence provides the strongest support for the hypothesis that (B)IAT effects are mainly driven by task-switching processes [48], which involve the ability to switch attention between different tasks, being less pronounced when associated categories share a response key. Unfortunately, less is known about the processes underlying the OMT, the CRT [62] or I-PCQ [2]. However, task switching can almost certainly be ruled out because neither the OMT and CRT nor the I-PCQ require participants to switch between tasks when completing these measures. Hence, because the OMT, CRT and I-PCQ, on the one hand, and the (B)IAT, on the other hand, are most likely associated with different underlying processes, they will also most likely predict different aspects of someone’s behavior [19,54,64,65].

In addition, Olson and Fazio [68], and Slabbinck and colleagues [54] suggested that non-symbolic measures such as the IAT rather measure general associations with (broad) categories, whereas symbolic measures such as the PSE and OMT [69] rather assess summary evaluations of specific situations. Because the OMT, the CRT and I-PCQ scores are constructed as summary scores of evaluations of a series of specific situations or questions, one could expect that the these measurements are also more situation specific (i.e., tentative to the specific scenarios or questions that are required to be evaluated). All these arguments together strongly suggest that the implicit measures that are commonly used in Management research and the BIAT are complementary rather than interchangeable.

Moreover, pragmatically, the BIAT is associated with substantially lower development costs vis-à-vis the CRT or OMT, the latter requiring substantial time and effort [39,70]. Unlike the OMT and CRT, but similar to the IAT and to a lesser extent the I-PCQ methodology, developing a new BIAT is a straightforward and relatively fast process: readily adaptable scripts exist [55], research stimuli can be recycled from a vast body of IAT studies, BIAT applications are rapidly growing, and BIAT scores can be automatically calculated. Furthermore, Management research often requires quick and simple data collection methods [2,23]; otherwise, response rates will drop below acceptable levels, or access to respondents cannot be obtained altogether [42,71].

Hence, administration time is an important determinant for the selection of appropriate measurement methods in Management research. For example, in a study with managers, Kehr [42] used the Multi-Motive Grid (MMG) for the assessment of implicit motives [72]. The MMG is a questionnaire-based measure that is frequently used for the assessment of implicit motives. Compared to the PSE, the MMG is easier and faster to administer, allowing data collection in applied field settings. The MMG, thus, seemed a good alternative to the PSE as pilot studies with managers showed unacceptable drop-out rates on the PSE. However, Schultheiss and colleagues [73] showed that measures such as the MMG tend to converge with explicit motives measures, and thus fail to capture important aspects of traditional implicit motives measures. As a result, many Management researchers for whom time-efficient data collection and data access are crucial, do not measure implicit motives at all, or rely on inferior measurement instruments. In all, as a consequence, the number of implicit motives studies in the Management research domain is very limited indeed [2,3,23,74], including Entrepreneurship [26]. As mentioned before, a BIAT can be completed in little over a minute [55]. This is considerably shorter than the time needed to complete a CRT or OMT, or any alternative method.

In all, we believe that the BIAT offers clear psychometric and practical advantages over the other available alternatives. Indeed, the BIAT is flexible and easy to adminster, has favorable psychometric properties, and offers ample opportunities to design new versions probing into a wide class of different motives, personality traits, attitudes, stereotypes, leadership and management styles, and ethical and societal dilemmas. This makes the BIAT an ideal candidate for measuring implicit motives in Management research. Of course, as for every new explicit and implicit measure, each new BIAT application needs to be thoroughly validated independently, and researchers must be and stay aware of the limitations of the BIAT. For example, the BIAT yields relative scores (e.g., the strength of the association between ‘sunshine and pleasant’ relative to the associative strength between ‘rain and unpleasant’) rather than absolute scores (e.g., sunshine is pleasant, regardless of someone’s attitude toward rain), which may limits its applicability, and its discriminatory and predictive power (for an intersting discussion, see [75–77]).

Below, we will apply our newly developed implicit motives BIAT in the context of an examination of entrepreneurial self-efficacy, business founding, and financial profitability. This endeavor serves two purposes. First, in so doing, we introduce a new data collection method in Management research, exploring its predictive validity in an entrepreneurial setting. Second, we hope to convincingly showcase that the study of implicit motives indeed offers great substantive potential in Management’s (sub-)disciplines, more broadly. Specifically, we will reveal that explicit and implicit motives have differential effects on important entrepreneurial attitudes, behaviors and outcomes. To us, this implies that the introduction of implicit, next to explicit, motives offers ample opportunities for new motives research in Management-related field settings.

### Implicit motives and entrepreneurship

So, the aim of our Entrepreneurship study is twofold. The first objective is to introduce the BIAT for the measurement of implicit motives. This is important because the availability of an easy-to-develop, easy-to-use and valid–and hence ‘appropriate’–measure could stimulate implicit motives research (and research into implicit attitudes, biases and traits, for that matter), especially in Management research where quick and easy data collection is crucial. Our second aim is to test BIAT’s predictive validity in a substantive study by focusing on the well-established observation that implicit motives predict long-term behavior and performance, whereas explicit motives are related to self-reflective appraisals, judgments, deliberate choices and decisions [31]. The selection of the criterion variables, and the underlying theoretical logic, is introduced below, leading to three hypotheses.

Firstly, the way in which business outcomes are measured is crucial. As long as business outcomes are captured by means of self-reported ‘subjective’ measures, we expect that primarily *explicit* motives profiles will predict these business outcomes, as then the explicitly self-reported entrepreneurial measures can be expected to correlate with the explicitly self-reported motives. Yet, if business outcomes are measured by means of ‘real’ or ‘objective’ (e.g., performance) metrics, measured through another source than self-reports (e.g., entrepreneurial ventures’ annual reports), then *implicit* motives measures should outperform their explicit counterparts [19,24,78].

*Hypothesis 1 (H1)**: (a) Explicit motives are related to self-reported ‘subjective’ business outcome measures, whilst implicit motives are not; and (b) Implicit motives are related to ‘objective’ business outcome measures, whilst explicit motives are not*.

An interesting entrepreneurship construct that can be taken as the ‘subjective’ business outcome dependent variable to explore this line of logic is the self-reported measure of Entrepreneurial Self-Efficacy (ESE) [79]. ESE is a highly relevant variable in Entrepreneurship research. ESE refers to the belief that one is able to perform activities required to start a business. ESE is a key determinant of entrepreneurial intention [80], venture creation [81], and entrepreneurial success [82]. ESE is found to be positively related to self-reported measures of need for achievement [83]. Regarding ‘objective’ business outcome measures, as we will explain below, we decided to use proxies for business founding and financial profitability, both on the basis of firm-level annual report data.

Secondly, the extensive literature reviews of Johnson [15] and Uhlmann [23] reveal that researchers in Entrepreneurship mainly focused on one particular motive: the need for achievement. Yet Murray compiled a list of 27 motives [27,28]. Because of their societal and economic relevance, Murray’s successors and contemporary motive researchers narrowed this lengthy motive inventory and drove the attention to three major motives: the power motive, the achievement motive, and the affiliation motive [31]. The power motive stems from a person’s desire to influence, teach or encourage others. Power-motivated individuals obtain satisfaction from exerting social, physical or emotional impact on others or on the world at large, but they experience social defeats and impacts from others as aversive [36]. People with a high need for achievement typically get satisfaction from mastering challenging tasks on their own, but experience the failure to master such tasks individually as dissatisfying [25].

Finally, people who are motivated by affiliation instead prefer to spend time with others they like. They love to create, maintain, and restore social relationships. They enjoy being a part of a group and have a desire to feel loved and accepted. Signals of rejection or hostility are experienced as unpleasant [84]. Interestingly, many aspects of business and career success have been linked to implicit motives. For example, McClelland and Franz [85] found that achievement-motivated individuals are less successful in jobs that require social skills because they are keen to function in an autonomous manner [25]. Similarly, results of McClelland and Boyatzis [86] indicated that, contrary to achievement-motivated employees, power-motivated employees strive for high-level positions in organizations so that they can control the direction in which their company is moving.

Tying these insights together, we expect that different implicit motives will become important at different developmental stages of a business’ lifecycle. Specifically, because many, if not most, companies start as micro-businesses, employing no or only a few people and with the ideas and skills of the entrepreneur being the most valuable [87,88], we expect that the achievement motive will drive business performance during the first years of a business. However, as the micro-business grows and employs more people, social skills become increasingly important [89]. In addition, as the growing business employs more people, not only the need to control the behavior of others increases, but also the opportunities that provide satisfaction to power-motivated entrepreneurs increase. Hence, growing businesses become ideal habitats for entrepreneurs who score high on need for power. So, the degree to which the entrepreneur is motivated by power will determine further business performance, rather than the entrepreneur’s level of achievement motivation.

*Hypothesis 2 (H2)**: (a) During the first years after the venture’s founding, implicit need for achievement is stronger related to business performance than implicit need for power; and (b) during later years after the venture’s founding, implicit need for power is stronger related to business performance than implicit need for achievement*.

Thirdly, business outcomes can take many different forms and shapes. As implicit motives orient, select and energize behavior [31,90], we expect that entrepreneurs will strive for goals that are congruent with their implicit motives. Power-motivated people tend to derive pleasure from their ability to have a physical, mental or emotional impact on others [91]. One of the consequences is that power-motivated people tend to engage in activities that increase their social visibility [91] and that provide a feeling that one is in control over her/his own life [21]. Therefore, on the one hand, we hypothesize that power-motivated entrepreneurs have founded *multiple* businesses because this provides expanded opportunities to increase their social visibility. The activities, behaviors, and outcomes associated with the venture-creation process also reinforce the entrepreneur’s notion that s/he is the master of her/his own fate [92,93]. On the other hand, as affiliation-motivated people prefer to spend time with others they like and love to maintain and restore social relationships [84], we expect that affilation-motivated entrepreneurs will mainly put their effort in a *fewer* businesses, trying to optimize social ties within one company before establishing another enterprise. Also, striving for good inter-personal relationships with their co-workers and customers, and placing long-term goodwill over short-term gain are competencies that distinguish average entrepreneurs from enterpreneurs who managed to turn an average business into a successful venture [94]. As these competencies are also characteristics of affiliation-motivated people [21], we expect that affiliation-motivated entrepreneurs will prefer to develop and expand fewer businesses.

*Hypothesis 3 (H3)**: (a) Power-motivated entrepreneurs have founded more businesses; and (b) Affiliation-motivated entrepreneurs have founded fewer businesses*.

## Method

### Design and participants

Our entrepreneurial sample consists of 108 Belgian entrepreneurs who participated in two on-line surveys and a series of workshops organized in Antwerp and Namur on entrepreneurial motives. Seventy-nine entrepreneurs are male (73%) and their mean age is 47.44 (*SD* = 8.65). From the first on-line survey, administered in 2012, we gathered self-reported measures on their objective performance (that is, the number of businesses the entrepreneur established) and a control variable (i.e., the age of the entrepreneur). Implicit motives and self-reported measures on the subjective performance of the entrepreneurs (that is, entrepreneurial self-efficacy or ESE) were assessed during a series of workshops in the summer of 2013. After a short introduction, the participants were led to a computer room where they were provided with three implicit motives BIATs [12] measuring the relative strength of (a) the power vis-à-vis the achievement motive, (b) the power vis-à-vis the affiliation motive, and (c) the achievement vis-à-vis the affiliation motive.

The order of the three BIATs was randomized, and the self-reported outcome measures were administered immediately after the assessment of the implicit motives. Survey data about the explicit motives profiles from the entrepreneurs were collected online in 2013, which is two to three months before the workshops, as part of the enrolling process. The information collected during the workshops was later matched with objective business outcome measures and the founding year of the business. We obtained this data from the Bel-first database containing Belgian annual report information. So, we have data from four distinct (and differently timed) sources: two online surveys, an assessment exercise (workshops), and an annual report database.

### Ethical statement

In Belgium and at the faculties to which the authors were affiliated with at the time that the data were collected, there were no requirements to obtain formal approval from an institutional review board (IRB) for non-clinical research studies, except when researchers might believe an approval is necessary (which was not the case here). Yet, the project, including the design of this study, was reviewed and monitored by the Federal Science Policy Office of the Belgian Federal Government (Belspo) and a Belspo-appointed project-specific advisory board. Belspo supports the Code of Ethics for Scientific Research in Belgium. All participants gave online (online survey) or verbal consent (exercises during workshop). Verbal informed consent for the workshops was viewed as sufficient as participants were not exposed to risky events, and all data were analyzed and stored anonymously.

Participants were also well informed of the purpose of the study. They were assured that no harm would come to them in the process of exercises, and were told that the exercise involved either the completion of questionnaires (online survey) or sorting tasks (exercises during workshop). Participants were also well aware and agreed that the information collected would be matched with measures that we obtained from other databases (such as the Bel-first firm accounts database). The results of all tests were kept private and participants were informed that they had the right to quit at any time during the completion of the survey or exercises. Participants were instructed to indicate their agreement to continue with the study by clicking forward after reading the informed consents (online survey) or by entering the computer room to start the sorting exercises (workshop) (see S1 Appendix for a detailed explanation of the data-collection process).

### Measures and procedures

#### Explicit motives: PRF

We adopted the affiliation, the dominance and the achievement sub-scales of the Personality Research Form (PRF) [95] to assess explicit motives. The PRF is a self-report inventory of motivational needs that is commonly used to assess explicit motives [73]. Each sub-scale consists of 16 statements, with participants indicating to what extent each statement suits them on a five-point Likert scale with anchors from 1 = “Fits not at all” to 5 = “Fits very well”. Sample items of the sub-scales are: (dominance) “The ability to be a leader is very important to me”; (affiliation) “I try to be in the company of friends as much as possible”; and (achievement) “I will not be satisfied until I am the best in my field of work”. For each sub-scale, we calculated the individual measures as the mean score of the scale items with high scores indicating a good fit between the motive and the participant. All sub-scales showed good to very good internal consistency (*PRF dominance*: *α* = .89; *M* = 3.59; *SD* = .64; *PRF affiliation*: *α* = .87; *M* = 3.98; *SD* = .59; *PRF achievement*: *α* = .80; *M* = 3.63; *SD* = .83).

The participants completed the measures in their native language. Because Belgium is a bilingual country, PRF items were presented in Dutch or French. We obtained the English and validated French version of Sigma Assessment Systems. Their version was translated into Dutch by a team of native Dutch speakers. The translated statements were discussed with researchers who had experience with PRF assessments in order to guarantee that the items reflect the true meaning of the original items.

#### Implicit motives: BIAT

We relied on the procedures of Slabbinck and colleagues [52–54] and Sriram and Greenwald [12] for the construction of the implicit motives BIAT. That is, we applied the instructions of Sriram and Greenwald [12] for the construction of the BIAT procedure, and adopted the recommendations of Slabbinck and colleagues [52–54] for the selection of the stimuli. The BIAT procedure consisted of seven blocks that allowed us to measure implicit power, implicit affiliation, and implicit achievement. The first block of the BIAT is a practice block, and the remaining six blocks capture the critical responses that were used to construct the BIAT scores. Critical blocks were presented in a random order. Each implicit motive involves two critical blocks, and each critical block consists of 20 trials. Table 1 presents an overview of the composition of the BIAT procedure. The full BIAT, including detailed instructions, can be downloaded from [reference withheld].

**Table 1:** Structure of the implicit motives BIAT and construction of the BIAT scores.

	Label	Label	Stimuli representing the …			
	Focal concept	Focal attribute	Focal concept: Pictures	Focal attributes: words	Non-focal concept: Pictures	Non-focal attributes: words
Practice block	—	Pleasurable	—	nice, friendly, pleasant, lovely		creepy, nasty, annoying, undesired
Critical block 1	Affiliation	Pleasurable	affiliation		power	
Critical block 2	Affiliation	Pleasurable	affiliation		achievement	
Critical block 3	Power	Pleasurable	power		affiliation	
Critical block 4	Power	Pleasurable	power		achievement	
Critical block 5	Success	Pleasurable	achievement		affiliation	
Critical block 6	Success	Pleasurable	achievement		power	

BIAT_pow-ach_: performance on critical block 4 versus performance on critical block 6

BIAT_pow-aff_: performance on critical block 3 versus performance on critical block 1

BIAT_ach-aff_: performance on critical block 5 versus performance on critical block 2

While completing the critical blocks, participants were requested to focus simultaneously on two labels. One label represented the focal concept (e.g., “Affiliation”) and the other label the focal attribute (e.g., “Pleasurable”). The labels appeared one under the other on the top center of the computer screen. Stimuli that were representative for the focal concepts plus focal attributes were presented one at a time on the center of the computer screen. Participants were required to categorize them as quickly as possible with one response key (e.g., the “i” key). Stimuli that were not representative for the focal concepts and attributes were also presented on the center of the computer screen, and participants were required to categorize them as “not belonging to the focal concepts and attributes” using another key (e.g., the “e” key). The practice block consisted of 20 trials. The structure of the practice block was identical to that of the critical blocks, with the exception that only the label of the focal attribute was provided (i.e., “Pleasurable”) and that participants were only required to categorize stimuli of the focal and non-focal attributes.

We followed Slabbinck and colleagues [52–54] regarding the selection of stimuli. For the target concepts, we used (Dutch or French translations of) the labels “Affiliation”, “Power”, and “Success”; for the attribute category, we took “Pleasurable”. The selection the attribute categories fits well with McClelland’s conceptualization of implicit motives, who defines ‘*a person with a strong motive as a person who has a strong* *affective* *response to an incentive*’ [9] and who argues that motives are based on affective experiences [31]. In a similar vein, Slabbinck and colleagues [52] demonstrated that IATs with affective attribute categories outperformed implicit motives IATs with attributes categories referring to the self (‘me’ vs ‘not-me’). The results of Slabbinck and colleagues [52–54] further indicated that motives can best be represented by pictorial rather than verbal stimuli. According to Gschwender and colleagues [96], pictorial stimuli enclose more concept-relevant information than verbal stimuli, which makes pictorial stimuli better suited for the assessment of traits such as motives. In addition, it is assumed that implicit motives are based on nonverbal experiences that are acquired early in life, before the development of language skills [97]. Because of this, nonverbal/pictorial stimuli that relate to implicit motives may be particularly suitable for capturing implicit motives. This is also shown by Slabbinck and colleagues [52], who tested different IAT variants for the assessment of implicit motives. They demonstrated that IATs with pictorial stimuli were superior to IATs with verbal stimuli.

Accordingly, the stimuli representing the target categories featured pictures demonstrating affiliation settings (e.g., kids walking hand in hand on the beach), power contexts (e.g., business man standing up straight at a meeting table), or achievement situations (e.g., a student who graduates and shows his degree). We had four pictures for each target category. For the attribute categories, we employed (Dutch or French translations of) “nice,” “friendly,” “pleasant,” and “lovely” to designate attractive, whereas we used “creepy,” “nasty,” “annoying,” and “undesired” to represent not attractive. We obtained all research materials from Slabbinck and his colleagues, who had carefully and systematically pretested all materials [52–54].

BIAT scores (e.g., the BIAT score of the power relative to the affiliation motive) were derived from the comparison of participants’ performance in two blocks that presented the same focal attribute (e.g., “Attractive”) but with two different focal concepts (e.g., “Affiliation” and “Power”). In total, we calculated three BIAT scores: that is, a BIAT score for the power relative to the achievement motive (*BIAT*_*pow-ach*_), a BIAT score for the power relative to the affiliation motive (*BIAT*_*pow-aff*_), and a BIAT score for the achievement relative to the affiliation motive (*BIAT*_*ach-aff*_). Thus, a high score on, for example, the BIAT_pow-ach_ indicates that the participant found it easier to pair power stimuli with pleasurable words and achievement stimuli with non-pleasurable words (see Table 1 –critical block 4) than power stimuli with non-pleasurable words and achievement stimuli with pleasurable words (see Table 1 –critical block 6).

Data from all critical blocks were used to compute BIAT scores, according to the scoring recommendations of Nosek and colleagues [55]. Extreme latencies below 400 milliseconds and above 2000 milliseconds were recoded to these boundaries, and the first two trials of each block were discarded. Individual BIAT scores were computed using the *D-*measure [98]. The *D*-measure was computed as the difference between mean latencies of the two critical BIAT blocks divided by the standard deviation of latencies in the those blocks [55]. Following standard procedures for the estimation of the internal consistency of latency measures [52–54,99], we estimated internal consistency of the BIATs by dividing each critical block into two sub-blocks of equal length. The first sub-block contained the even trials and the second the odd trials, and BIAT scores were calculated for each sub-block separately. The Spearman–Brown coefficients revealed a good split-half reliability for all three BIATs (*BIAT*_*pow-ach*_: .79 ;*BIAT*_*pow-aff*_: .77; *BIAT*_*ach-aff*_: .78). Participants required, on average, 3.45 minutes to complete the three BIATs (*SD* = 1.55).

#### Entrepreneurial self-efficacy (ESE)

Inspired by McGee et al. [100], and Zhao et al. [80], we measured ESE in terms of the subjects’ confidence in their ability to perform critical entrepreneurial tasks, including: (1) searching for opportunities; (2) creating new products; (3) thinking creatively; (4) commercializing ideas and new products; (5) fund raising; (6) selling new products or services; (7) solving other people’s problems; (8) finding new ways to solve problems; (9) imagining different ways of thinking and doing; and (10) creating artistic value. Items were measured on a seven-point Likert scale, ranging from 1 (“much worse than fellow entrepreneurs”) to 7 (“much better than fellow entrepreneurs”). The original scale items were in English and translated into Dutch and French, following the same procedure as for the PRF items (see S2 Appendix for all items).

In the literature, consensus as to ESE’s dimensional structure is lacking: e.g., Chen et al. [79] suggest a five, Kickul et al. [81] a four, and Zhao et al. [80] a one-dimensional structure. In our case, factor analysis reproduced three dimensions, which can be referred to as “Growth and expansion” (items 1 to 4; *EV* = 2.55; *α* = .77), “Problem solving” (items 5 to 7; *EV* = 2.18; *α* = .75), and “Creativity” (items 8 to 10; *EV* = 1.88; *α* = .62). Scores were averaged over all items to construct an individual measure of each of ESE’s dimensions. Following Zhao and colleagues [80], averaging over all items gives a total ESE measure, with a good internal reliability of *α* = .80 (*M* = 4.80; *SD* = .76). As we have no theoretical prior as to differential effects of ESE’s sub-dimensions, our main analyses report the results for total ESE. In exploratory analyses, we estimated full models for each sub-dimension separately (available upon request).

#### Objective business outcomes

Objective entrepreneurial outcomes are measured with two metrics. First, we include the number of businesses each participant established in her or his life. Second, based on the VAT number of the participant’s principal business, we merged our entrepreneurial sample with the Bel-first database, containing financial performance data on most Belgian companies in order to obtain the gross profits of the participant’s principal business of the two most recent fiscal years that were available at the time (i.e., fiscal years 2012 and 2013). The gross profit is the difference between revenue and the cost of making a product or providing a service, before deducting overhead, payroll, taxation, and interest payments. Gross profit is a good measure to study true output/market performance, because the sums that are taken into account for overhead, payroll, taxation, and interest payments may depend on financial or strategic decisions that are not directly connected with market performance (e.g., tax optimization versus optimization of shareholder value). For the same reasons, we also believe that it is better to study absolute financial performance measures rather than relative indices (e.g., such as ROI and ROA) because many of these indices relate equity measures (e.g., net income) to non-equity measures (e.g., total assets for ROA, and net investments for ROI). As non-equity measures may be driven by other decision processes (e.g., marketing decisions particularly influence income levels, whereas financial decisions such as depreciation rates especially influence total level of assets), financial indices are less suited to test our hypotheses.

Because six companies provided incomplete or erroneous VAT numbers and 17 enterprises were not listed in the Bel-first database, we could only build our model on financial performance data of 85 enterprises. To test for selection bias, we compared the major variables (implicit and explicit motives, ESE, number of established companies, age of entrepreneur, et cetera) for enterprises that did and did not provide financial performance data. With *t*-values ranging from .19 to 1.57, we found sufficient evidence to conclude that selection bias is not a serious problem.

#### Control variables

Since business outcomes are contingent upon the age of the enterprise, the age of the entrepreneur and the size of the enterprise, we included the entrepreneur’s age, the year of establishment of the participant’s principal business, and the company’s paid capital as covariates [87] (see S2 Appendix for the survey questions). We subtracted the year of establishment from the year of the motives measurement, 2013, to obtain the age of the enterprise. We apply a natural log transformation to variables that are highly skewed, clustering toward either side of the distribution, with a long tail. Descriptive statistics are given in Table 2, indicating that multicollinearity is not an issue.

**Table 2:** Descriptives, α coefficients, and correlations of all dependent and independent variables.

		1.		2.		3.		4.	5.		6.	7.	8.		9.		10.		11.		12	13
1.	PRF dominance	(.89)																				
2.	PRF affiliation	0.20	*	(.87)																		
3.	PRF achievement	0.52	**	0.31	**	(.80)																
4.	BIAT pow-ach	-0.28	**	0.06		-0.19	*	(.79)														
5.	BIAT pow-aff	-0.16		-0.11		-0.08		-0.10	(.77)													
6.	BIAT ach-aff	0.13		0.19		-0.02		-0.06	-0.01		(.78)											
7.	ESE	0.34	**	0.21	*	0.34	**	-0.13	0.01		0.09	(.76)										
8.	Number of businesses	0.15		-0.13		-0.09		-0.05	0.27	**	-0.04	0.12	—									
9.	Capital (X1.000)	0.16		-0.08		-0.09		0.16	0.03		0.16	0.15	0.08		—							
10.	GP 2012 (X1.000)	0.29	*	-0.04		0.08		0.12	0.16		0.11	0.09	0.24	*	0.26	*	—					
11.	GP 2013(X1.000)	0.22		-0.09		0.09		0.09	0.21		0.04	0.14	0.25	*	0.19		0.94	**	—			
12	Age of entrepreneur	0.15		0.04		0.18		0.06	-0.08		-0.13	0.12	0.09		-0.01		0.01		0.07		—	
13	Age of enterprise	0.15		0.19		0.15		0.14	0.01		-0.08	0.13	0.12		-0.02		0.55	**	0.49	**	0.20	—
	M	3.59		3.98		3.63		-0.16	-0.29		-0.06	4.80	3.26		196		316		274		47.44	18.46
	SD	0.64		0.59		0.83		0.85	0.84		0.83	0.762	2.30		370		572		529		8.65	11.31
	Skewness	0.01		-0.33		-0.27		0.02	0.40		0.17	-0.32	4.67		2.91		2.47		2.37		-0.01	2.70
	Kurtosis	-0.4		0.83		-0.04		-1.44	-1.09		-1.41	-0.16	32.98		8.50		6.06		5.40		0.30	11.25

Coefficient α estimates are presented in parentheses; GP = gross profit

* *p* < .05 and

** *p* < .01.

Two remarks are worth making. A first comment relates to the absolute vis-à-vis relative nature of our measures, given the debate in the literature regarding difference scores [Edwards, 2001]. What we do here is the standard way of treating PRF measures and comparing them with implicit motives scores [22,51,54,101]. Thus, transforming the absolute PRF measures into relative PRF measures would make our study less comparable with other implicit motives research. In addition, Schultheiss et al. [73] demonstrate that implicit and explicit measures are measures of distinct constructs, and that correlations between measures of both constructs are not affected by the methods with which both constructs are measured, even if the methodological correspondence between the explicit and implicit measures is maximized. Further, the independence of implicit and explicit motives, regardless of the way in which they are measured, has recently been confirmed in a meta-analysis [8]. Indeed, our correlations between implicit and explicit motives are not affected by the method that is used to calculate motive scores. By way of robustness check, we calculated relative PRF measures in a similar way as the BIAT scores were constructed (i.e., subtracting the average score of a PRF scale from the average score of another PRF scale, divided by the standard deviation of all the items of those scales). Inspection of the bivariate correlations (available upon request) shows that the usage of relative instead absolute PRF measures does not affect the magnitudes of the correlations between implicit and explicit motive measures.

A second comment involves the timing of our measurements, and what this implies for causality claims [102]. To cope with the postdictive nature of our dependent variable, we linked our entrepreneurial sample with the Bel-first database. By doing so, we obtained financial performance data of 85 companies for the years 2012 and 2013. We realize that this approach does not fully resolve the issue of the postdictive nature of our dependent variable (the motives of the entrepreneurs were assessed in the middle of 2013]. However, because (implicit) motives and personality traits seem to evolve only slowly in adulthood [103–106], we believe it is legitimate to conclude that our results are reliable and valid. Of course, we acknowledge that making causal claims would not be warranted. Thus, instead of talking about ‘determinants’, which imply causality, we refer to associations between motives and business performance.

## Results

### Explicit motives predict declarative behavior

We estimated an ordinary least squares regression model with total entrepreneurial self-efficacy (total ESE) as a function of implicit motives and explicit motives. To assess the relative importance of implicit and explicit motive measures in predicting behavior, we performed of relative weights analysis [107,108]. Relative weights analysis transforms a set of predictors into a new set of orthogonal variables that are as highly correlated to the original set of variables. The dependent variable (i.e., total ESE) is then regressed on the new uncorrelated predictor variables. To compute estimates of relative importance, these coefficients are then squared and combined with the squared standardized regression coefficients obtained by regressing the original variables on the orthogonal variables. Finally, the raw relative weights are transformed into proportions by dividing them by the sum of all raw relative weights, yielding a proportional contribution of each original variable [108,109]. In addition to the relative weights, we used a bootstrapping procedure with 1,000 bootstrap samples to construct confidence intervals for the relative weights, following Johnson’s procedure [110]. The results are summarized in Table 3.

**Table 3:** Implicit motives and total entrepreneurial self-efficacy (ESE).

	*B*	*SE*	*p*	*VIF*	*Relative weights (95% CI)*	
Constant	1.79	0.65	0.01	—	—	—
PRF dominance	0.37	0.17	0.03	1.52	39%	(9%, 60%)
PRF affiliation	0.07	0.12	0.57	1.14	14%	(1%, 38%)
PRF achievement	0.39	0.17	0.03	1.49	39%	(10%, 61%)
BIAT _pow-ach_	0.04	0.09	0.67	1.14	4%	(1%, 25%)
BIAT _pow-aff_	0.08	0.08	0.36	1.06	1%	(0%, 23%)
BIAT _ach-aff_	0.09	0.08	0.28	1.05	3%	(0%, 22%)

Dependent variable: entrepreneurial self-efficacy.

*R*^2^ = .19.

The model explains 19 per cent of the variation in total ESE (*p* < .01). The regression results show that none of the implicit motives are significantly associated with total ESE (all *p*’s > .05). Yet, the results further yield significantly positive parameter estimates for explicit need for achievement (*b* = 0.39; *p* < .05) and explicit need for dominance (*b* = 0.37; *p* < .05). Explicit need for affiliation (*b* = 0.07; *p* = .57) is not predictive for total ESE. The PRF measures obtained the highest relative weights, and these weights are in line with the parameter estimates. Even though confidence intervals of the relative weights of PRF and BIAT measures overlap, explicit motives explain more than 90 per cent of the variance in total ESE, compared to eight per cent for the implicit motive measures. These findings are in line with previous literature and offer support for H1.

In exploratory analyses, we estimated the above ESE models for all three sub-dimensions separately (full tables are available upon request). In line with the above, we find that none of the BIAT measures is significantly related to any of the ESE sub-dimensions and that the relative weights of the BIAT measure are always considerably lower than those of the PRF measures. Moreover, explicit need for affiliation is non-significant, as for total ESE, except for the “growth and expansion” sub-dimension. Explicit need for achievement co-predicts all three sub-dimensions, explicit need for dominance does so for “Growth and expansion” and “Problem solving”, and explicit need for affiliation for “Growth and expansion”. Again, as in the case of total ESE, this pattern of results is fully in line with H1.

### Implicit motives predict objective business outcomes

**Fig 1:**
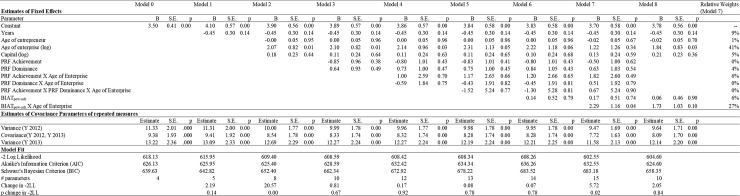
Implicit motives profiles and business performance (gross profit).

First, in order to examine the relationship between implicit motives and objective financial profitability, we analyzed our data by means of repeated measures with linear mixed models. The log of gross profit was treated as the repeated dependent variable, and time as a repeated independent variable. Before examining the relationship between implicit motives and gross profit, we first estimated an unconditional model (with no predictors involved) and found significant variation in the time variable (Fig 1). This confirmed that a repeated-measures mixed model was the correct data analysis technique to use. We estimated seven hierarchical models to test whether the implicit achievement motive predicts objective financial profitability (log of gross profit) during the first years of a business, whereas the power motive is predictive for later objective financial profitability.

In Model 1, we added time as a fixed variable. In Model 2, we entered the control variables (age of entrepreneur, log of age of enterprise, and log of capital). The measures of explicit power and achievement motives were entered in Model 3, and their interactions with the log of the age of the enterprise were included in Model 4. The three-way interaction between the explicit power measure, the explicit achievement measure, and the log of the age of the enterprise was added in Model 5. The measure of the implicit power versus achievement motive was entered in Model 6. The interaction between the measure of the implicit power versus achievement motive and the log of the age of the enterprise was included in Model 7. Finally, we also estimated a model that only included the control variables, together with the implicit power versus achievement motive, the log of the age of the entrepreneur, and the interaction between the implicit power motive and the log of the enterprise’s age (Model 8).

We grand mean-centered all of the independent variables to facilitate interpretation [111] and used − 2 log likelihood (− 2LL) and BIC to assess model fit. To assess the incremental fit of our models, and hence the predictive validity of our measures, we evaluated the significance of the change in − 2LL from one model to the next. To do so, we performed a series of chi-square tests with the degrees of freedom equal to the difference in the number of parameters for the pair of nested models. We also inspected the evolution of the BIC as this measure includes a penalty function that is based on the number of estimated parameters. Additionally, we performed a relative weights analysis on Model 7 to evaluate the relative importance of explicit and implicit motive measures in predicting business performance. The results of these analyses are presented in Fig 1.

The incremental fit statistics (change in -2LL) in Fig 1 show that neither the addition of explicit motive measures, nor their interaction with the age of the enterprise did improve model fit. This is in line with H1b. However, the -2LL statistic improved significantly when the interaction between the implicit motive measure of power versus achievement and the log of the age of the enterprise was entered to the model. Removing explicit power, explicit achievement and their interactions with the log of the age of the enterprise did not deteriorate model fit (Model 8 vs Model 7) and fitted the data marginally significantly better than a the model that only comprised the control variables (Model 8 vs Model 2: Change in -2LL: 4.80; *p* = .091). Inspection of the BIC values further reveals that inclusion of explicit motive measures increased the BIC values (Model 3–5) and that BIC only decreased when the interaction between the implicit motive measure and the log of the age of the enterprise was entered into the model (Model 7 vs Model 3–6). However, the BIC value of Model 8 was higher than the BIC value of the Model 2 (model with control variables only). The parameter estimates of Model 7 show that older enterprises are more profitable than younger enterprises. Yet, more importantly, this effect is qualified with a significant interaction with the implicit motive measure.

**Fig 2:**
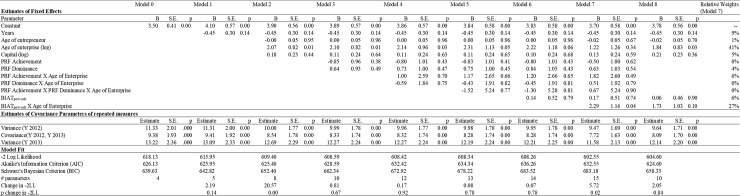
Johnson-Neyman plot of the region of significance for the effect of implicit power versus achievement on gross profit across the life span of an enterprise.

To analyze this interaction in depth, we performed a Johnson-Neyman analysis [112]. This technique allows us to estimates the conditional effect of the implicit power versus achievement motive for different age levels of the enterprise and identifies regions of significance. For ease of interpretation, we do this plot for the non-logged age of the enterprise. Robustness analyses revealed that the results presented above are similar if we use non-logged rather than logged age of the enterprise (available upon request). These regions indicate for which ages of the enterprise the entrepreneur’s implicit power versus achievement motive has a significantly positive, non-significant, or significantly negative impact on the enterprise’s gross profit. Fig 2 illustrates the relation between the age of the enterprise and the impact of the entrepreneur’s implicit motive profile on the enterprise’s performance. The figure shows that companies grow (marginally) faster in the first six years after establishment when their entrepreneur is implicitly motivated by achievement rather than by power (age of enterprise = 6.41 years: *b* = -1.95; *p* = .10). The gross profit of mature companies seems to not to be affected by the entrepreneur’s implicit power and achievement motives as it takes up to 26 years after establishment before gross profit starts benefitting from an entrepreneur with a dominant implicit power motive (age of enterprise = 26.5 years: *b* = 1.49; *p* = .10).

Altogether, the fit statistics, the parameter estimates and the relative importance of the predictors indicate that it is not worth adding explicit motive measures to explain business performance, whereas most evidence shows that implicit motives do explain an important part of an enterprise’s business performance. This largely confirms our H2a and H2b.

Second, to test H3a and H3b that implicit power-motivated entrepreneurs have founded multiple businesses, whereas implicit affiliation-motivated entrepreneurs mainly put their effort in a single business, we analyzed the relationship between the log of the number of businesses each entrepreneur established in her or his life and her or his motives profiles, using an ordinary least squares regression model with heteroscedastic consistent standard error estimator (HC3). We conducted three regression models. In the first model, we regressed the logged number of businesses on the age of the entrepreneur, and the PRF dominance and PRF affiliation scores. In the second regression model, we entered the interaction between both PRF measures. Finally, in the third model, we entered the *BIAT*_*pow-aff*_ score and compared the incremental validity of this model by assessing the significance of the change in *R*^2^ from the second to third model. We also performed a relative importance analysis on the parameter estimates of the last model.

Table 4 contains the regression coefficients, the fit statistics of the estimated regression models and the relative importance weights of the parameter estimates The results show that none of the explicit motive measures reached significance (all *p*’s > .05) and that explicit motives were relatively unimportant in predicting the log of the number of businesses each entrepreneur established, which again is in line with H1b. Consistent with our prediction, the significant parameter of the *BIAT*_*pow-aff*_ score and its high relative importance weight (68%) indicate that entrepreneurs who are implicitly motivated by power rather than by affiliation established more businesses than entrepreneurs who are implicitly motivated by affiliation rather than by power (*b* = .82; *p* < .05). In addition, the significant change in *R*^2^ from Model 2 to Model 3 established the incremental validity of the *BIAT*_*pow-aff*_ in predicting this aspect of business performance.

**Table 4:** Implicit motives and number of established businesses.

	Model 1			Model 2			Model 3			*Relative weights (95% CI)*	
* *	*B*	*SE(HC)*	*p*	*B*	*SE(HC)*	*p*	*B*	*SE(HC)*	*p*		
Constant	-1.08	0.30	0.35	0.27	1.76	0.53	1.19	1.87	0.92	—	—
Age of entrepreneur	0.00	0.00	0.63	0.00	0.00	0.65	0.00	0.00	0.48	4%	(0%,40%)
PRF Dominance	0.11	0.08	0.18	0.34	0.54	0.53	0.07	0.57	0.90	16%	(1%,51%)
PRF Affiliation	-0.06	0.07	0.34	0.18	0.50	0.72	-0.13	0.54	0.81	5%	(1%, 27%)
PRF Dominance X PRF Affiliation				-0.07	0.15	0.64	0.02	0.16	0.89	7%	(2%, 24%)
BIAT_pow-aff_							0.14	0.05	0.01	68%	(10%, 89%)
*R*^2^	0.031			0.034			0.115				
Δ *R*^2^	0.031			0.003			0.081				
*F* Δ (*df*1,*df*2)	.768 (3,104)			0.215 (1,103)			6.131 (1,102)				
*p*	0.51			0.644			0.014				

## Discussion

Our results show that the BIAT is a good measure for the assessment of implicit motives, and that this is the case both from a psychometric as well as a practical viewpoint. In line with implicit motives theory [31,73], the data from our study provide initial evidence that the BIAT predicts the kind of behavioral patterns that are assumed to be determined by implicit motives (i.e., spontaneous behavior and long-term behavioral trends). Confirming the divergent validity of the BIAT, only explicit motives are related to responses in specific situations that require conscious thought and deliberation (i.e., entrepreneurial self-efficacy). In addition, the found relationships between explicit motive scores and the measures of entrepreneurial self-efficacy may be partially explained by socially desirable responding or common-method variance [102,113].

### Methodological implications

The BIAT has definitely a unique advantage that condones its use in Management research. That is, the BIAT substantially reduces the data administration and preparation time in comparison with other techniques such as OMT and PSE. In our study, it took 3.45 minutes to obtain reliable and valid measures of three implicit motives. If we had used three traditional IATs instead or if implicit motives had been assessed by means of the PSE or CRT, participants would have needed up to 20–30 minutes to complete these tests [12,33,52]. Data preparation of IAT and BIAT procedures is fully automated [12]; but in case, had we used the PSE, we would have spent up to 60 hours to prepare our data for analysis [33]. Moreover, we constructed new BIATs to measure the implicit needs for achievement, affiliation and power. These three BIATs all work well, and are ready to be used in other Management research, given their efficiency, reliability and validity.

BIAT’s ease of use and flexibility imply that new BIATs can be relatively easily developed and validated. Hence, the BIAT might help to overcome the lack of research into implicit constructs in areas where time-efficient data collection is crucial, of which studies in managerial and organizational settings are a key example. Indeed, we argue that the lack of Management research into implicit motives may well be due to the unavailability, to date, of an easy-to-administer and easy-to-develop measurement instrument, which is critical in the area of field work in the context of research involving difficult-to-access and busy target groups like employees, entrepreneurs and managers (different from the students routinely used in much psychology research, mostly in lab settings). However, the added value of the BIAT is not limited to its time efficiency. As we argued, the BIAT is a non-symbolic measure in contrast to most, if not all, other implicit measures that are currently used in Management research, being symbolic measures. As different types of measures may predict different types or facets of (business) behavior, and as method and theory inevitably interact [114], we believe that the use of the BIAT in Management research will bring novel insights in Management theory. We provide some ideas in the next section. We also argued that the BIAT is complementary to both explicit and other implicit measures from a theoretical perspective. Yet, future research needs to discover the unique contributions of the BIAT and other implicit measures in predicting business behavior. To do so, we encourage researchers to include different types of implicit measures in their research design, and to strive for a methodological fit between the selected research methods and the studied behavior. This type of research may yield more complete and holistic insights.

### Practical implications and future research

Our results have also important implications for research in different (sub-)disciplines in the broad domain of Management, from Organizational Behavior and Strategy to Accounting and Marketing, and many more. We speculate that the introduction of implicit constructs (such as implicit attitudes, biases, motives and traits) in Management research (including Entrepreneurship) has the potential to generate many novel insights. Our study shows that the entrepreneur’s implicit motives are important determinants of her or his venture’s success. Failing to do so would imply that Entrepreneurship research into motives could wrongly be considered to be a dead-end, and certainly would produce biased and incomplete insights.

Interestingly, for instance, our lifecycle-dependent motives logic might offer an explanation for a well-known puzzle in the Entrepreneurship literature: gazelles are very rare, and the *R*^2^ associated with gazelles studies is very low [115]. Gazelles are defined as entrepreneurial ventures that grow sustainably over a long period of time. H2 would predict that this is difficult to achieve because (a) different implicit motives are key during different stages of a venture’s lifecycle, and (b) such implicit motives evolve only slowly in adulthood [105,106]. H2 suggests that turning a successful start-up into a gazelle requires a switch in the dominance of implicit motives from achievement to power. An entrepreneur with both high implicit power and achievement motives may have the ability to shift focus as necessary without “changing” his or her personality. Yet, if the entrepeneur’s is only motived by achievement, such a shift is unlikely to happen, requiring other mechanisms to achieve the needed implicit motive shift. For example a replacement of an achievement-motivated founding entrepreneur by a power-motivated successor, or adding a power-motivated CEO to the venture’s founding team may be good strategies to turn promising start-ups into successful gazelles. Of course, also other variables such as creativity, work ethics, timing, and networking do also play an important role in entrepreneurial succes, and many of these variables may interact with or operate independently from the motives that serve as the focus of this paper. Regrettably, we cannot explore these potential explanation with our cross-sectional data. Future research may explore these lines of logic further.

As said, the applicability of the BIAT moves far beyond our illustrative example of implicit motives in Entrepreneurship research. Here, two further examples should suffice. First, implicit constructs fit squarely with the recent plea to develop a Behavioral Strategy literature. According to Powell et al. [116] (p. 1369), “Behavioral strategy applies cognitive and social psychology to strategic management theory and practice.” A clear example relates to upper echelon theory and top management team studies. Here, a key question revolves around the impact of deep-level characteristics of (teams of) top managers on strategic decision-making, of which implicit motives and traits are prime examples. Second, implicit constructs are important in the context of many Organizational Behavior issues. As Harms and Luthans [2] (p. 589) observe, “Implicit psychological constructs are effective predictors of behavioral outcomes but are rarely used in organizational settings because of real or imagined problems with measurement validity and administration.” They use the example of an implicit measure of psychological capital to illustrate that this current state of affairs can be turned around.

The study of implicit psychological constructs should not be limited to implicit achievement, power and affiliations motives, assessed by BIATs. As mentioned before, contemporary implicit motive research is rooted in Murray’s research on human motives. Murray believed that people share the same basic sets of 27 needs and differ only in their priority ranking of these needs [27,28]. Murray’s successors, and especially McClelland, Veroff, Heyns, and Atkinson [25,117,118], drove the focus on the achievement, affiliation, and power motives. Yet, this selection of motives was based on their personal interests, not on any theoretical ground or empirical evidence. Hence, even though the list of potential motives is, in all likelihood, limited to a small number of motivational systems [119], it may be worthwhile to go back to the roots of implicit motives research and study if other, still unexplored motives do exist from which Management research could benefit.

Additionally, future applications should also not be limited to BIAT studies. As we argued before, different types of implicit measures are most likely complementary and not interchangeable, tapping into different mechanisms and relating to different outcomes. Schoen and colleagues [59], for example, recently developed a CRT measure to assess a person’s implicit creative personality and found that it is a substantial predictor of creative performance. Yet, other implicit measures of creative personality, such as a BIAT, an OMT or a I-PSQ-type of questionnaire, could most likely add to the knowledge of creative personality and performance.

## Limitations

Of course, as any study, our work has limitations that point to promising future research avenues. First, although the present study provides initial support for the validity of the BIAT as a measure of implicit motives, generating convincing results in an entrepreneurship context, more work is required before the BIAT can be established as a valid alternative to the in-depth story-writing alternatives that are based upon detailed and fine-grained content-coding procedures. Specifically, more replications are needed, preferably with a variety of different outcome variables, in diverse research domains, including Management research, and with different types of participants. In this context, the BIAT instrument introduced above provides, we believe, a very promising tool that can be further developed and perfected in future work.

Second, correlations between implicit and explicit motives are in general not significant [8,120]. Yet, in our study, the correlations between the BIAT_pow-ach_ and PRF dominance as well as PRF achievement were significant and negative (see Table 2). One may argue that the BIATs in our study are not independent from explicit motive measures, and hence are no valid measures implicit motives. However, significant correlations between implicit and explicit motive measures are not uncommon. In fact, we believe it would be better to talk about ‘inconsistently related measures’ than about ‘unrelated measures’. To illustrate, Thrash et al. [121] show that implicit and explicit achievement are systematically related and suggest that (some) individuals may use implicit motives as a foundation for the development of explicit motives. This and other research [122–124] reveal that contextual and dispositional factors predict the degree of congruence between implicit and explicit motives. Hence, it may be that an (unobserved) contextual or dispositional factor accounts for the found significant correlation. Future research on the relation between implicit and explicit motives may further clarify the conditions associated with the congruence between implicit and explicit motives. Because of its ease of use, the BIAT may serve as an excellent tool for this purpose.

Third, we established correlational relationships between implicit motives measures and outcomes of (partially) past behaviors to test the ‘predictive’ validity of the BIAT (i.e., the number of established enterprises and approximately half of the gross profit was realized before the assessment of the motives profiles). Yet, correlations between implicit motives and behaviors could stem from past actions shaping implicit motives rather than implicit motives orienting future behaviors. For example, entrepreneurial failure or success may condition new automatic associations, and hence alter implicit motives profiles. Of course, a better way to examine the causal relationship between implicit motives and behaviors / outcomes, and hence to test the predictive validity of the BIAT, is to use a longitudinal design [102]. For example, one can examine whether implicit motives profiles assessed at Time 1 predict behaviors at Time 2 more effectively than behaviors at Time 1 predict implicit motives profiles at Time 2, pointing to a potential causal effect of implicit motives.

Fourth, a longitudinal design may also address the dynamic or malleable nature of some of our outcome measures. For example, the number of businesses started at Time 1 may influence the likelihood to start a new business at Time 2. Also, whenever an entrepreneur starts a new business, her or his (motivational) influence on the operations and profitability of her/his previously established businesses may alter. For example, it could be that power and achievement-motivated entrepreneurs will focus more on the new business because the challenging and uncertain character of a newly established company is rewarding for their motivational profile. However, this shift in focus may imply that the influence of their motivational profile on the operations of the previously established companies decreases. This dynamic may be different for affiliation-motivated entrepreneurs, who aim to maintain good and stable relationships with all employers in both previously and newly established companies. Yet, in absence of longitudinal BIAT data and given the very promising potential of introducing other implicit motives measures in entrepreneurship research, the design of the current study was the best we could offer.

Fifth, an experimental validation of the BIAT would be very worthwhile [125]. This approach tests if variations in implicit motives causally produce variations in the outcomes of the BIAT. As far as we know, this approach has not yet been applied to any BIAT measure. Closest comes Slabbinck et al. [52], who applied Borsboom et al.’s [91] approach to validate their implicit power IAT. They aroused implicit power in half of their participants and showed that the IAT effect was larger in the power-arousal condition than in the control condition. Of course, even though the BIAT procedure shares many properties with the IAT’s, we cannot simply claim that this finding also holds for the BIAT, but we believe that it is likely that similar effects would occur if we applied such a procedure to the BIAT. However, there were some practical considerations that hampered us from doing so in the context of the current study, and to leave this for future research.

One of the main goals of our study is to develop and validate an implicit measurement tool that is easily employable in Management research. Unfortunately, an experimental design in such a setting is not that evident. Therefore, we chose a rather pragmatic approach by focusing on the measure’s predictive validity. By doing so, our approach is rather in line with Newton and Shaw’s pragmatic view on measurement validity [126], arguing that measures should be primarily judged in relation to their measurement, decision-making and broader policy aims. We certainly will not go as far as Newton and Shaw, and believe that both Borsboom et al.’s [91] causal and Newton and Shaw’s [112] pragmatic approaches are needed to validated measures. But given the specificities of our sample and research goal, we believe that it was legitimate to start with our pragmatic approach, hoping that our first ‘successes’ may inspire and instigate future work to continue the further validation of our BIATs.

Sixth, we combined verbal stimuli that represented the attribute categories (i.e., pleasant versus unpleasant) with pictures that represented the concept categories. We also contrasted one motive (e.g., need for power) with another motive (e.g., need for achievement). This resulted in BIATs measuring the relative strength of the power vis-à-vis the achievement motive, the power vis-à-vis the affiliation motive, and the achievement vis-à-vis the affiliation motive. Of course, many other BIAT variants may be constructed, each with its own (dis)advantages. For example, instead of contrasting one motive with another, one could opt to contrast the approach dimension of a motive (e.g., achievement: hope for success) with the avoidance dimension of that motive (e.g., achievement: fear for failure). Such a BIAT would result in an absolute motives measures, but it will lose the property that motive-eliciting measures should be sufficiently ambiguous and should also provide some cues for other motives than the motive in question [34]. Thus, translating this into BIAT features, we thought it was best to contrast stimuli of one motive with stimuli of another motive. The advantage of this approach is that the results are better comparable with other types of implicit motives measures. However, the downside of our approach is that our measures are relative measures, and thus more difficult to interpret. Also, for the same reasons as why we chose to work with pictorial stimuli representing the motives, one could also opt to use visual stimuli such as emoticons or emotional manikins [127] as stimuli representing the affective attribute categories. These examples illustrate that more methodological research on the BIAT properties are needed.

Seventh, it is possible that our entrepreneurial sample suffered from self-selection bias. For example, long-standing affiliation-oriented entrepreneurs with multiple business may have chosen to avoid participating in the workshops to devote time to their multiple businesses to remain affiliated with their employees, customers, and clients. Such a confound is always a concern in studies that are not purely randomized and experimental in nature [102]. Thus, future research should explore the relationship between motives and business outcomes by means of other methods and research designs, including case studies and lab experiments.

Eighth, the content-coding methods may be labor-intensive and time-consuming, but they also offer some advantages over the BIAT. The BIAT requires cooperation of participants. However, content-coding systems can be applied to a wider range of material that is already available or that has not been collected for other research purposes [33,36]. For example, Winter’s [36] scoring system can be used to evaluate any kind of written or spoken material that is at least partially imaginative (e.g., speeches, blogs, interviews, or literary works). Moreover, content-coding systems enable researchers to measure implicit motives at a distance, which grants them access to a pool of otherwise unavailable data from respondents who might be deceased or live in remote locations. Also, motives researchers showed that any given implicit motive may not be a unitary construct, but rather could consist of different facets (e.g., achievement as fear for failure and hope for success]. Many direct motives measures and traditional implicit personality procedures allow researchers to score motives on their underlying facets. Perhaps a series of BIATs, each aiming at a specific facet of implicit motives, could provide an alternative to the flexibility and versatility of direct measures and traditional implicit motives measures. However, BIAT always require participants to cooperate, which limits the extent to which it can be implemented in labs or online.

## Conclusions

In conclusion, the BIAT is an easy-to-use and valid measure of implicit motives that has the potential to boost the introduction of implicit constructs in Management research. Given what we know from the psychology of motives, many subtle effects of motives on attitudes, behaviors and outcomes go unnoticed when Management researchers focus on their explicit manifestations only. After all, implicit motives orient different types of attitudes, behaviors and outcomes than explicit motives do. For example, as we revealed in our substantive example, implicit motives may affect entrepreneurial outcomes where their explicit counterparts do not, and vice versa. Of course, the validity of a measure cannot be established in one study [102], but rather requires looking at various sorts of validity over a series of studies. Hence, we hope this paper encourages Management scholars to apply the BIAT to achieve a superior understanding of the effects of (explicit *and* implicit) motives on a wide range of attitudes, behaviors and outcomes in many different managerial and organizational settings and contexts.

## Supporting information

S1 Appendix How did participants give their consent to take part of the study? (DOCX)

S2 Appendix Overview of survey questions. (DOCX)
