# Supplementary material for: The added value of implicit motives for management research Development and first validation of a Brief Implicit Association Test (BIAT) for the measurement of implicit motives
Source: PLoS One. 2018 Jun 20;13(6):e0198094. doi: 10.1371/journal.pone.0198094 (PMC6010206; doi:10.1371/journal.pone.0198094)
Supplement: S1 Appendix — (DOCX) [file pone.0198094.s001.docx]

**S1 Appendix. How did participants give their consent to take part of the study?**

* Participants participated in a large-scale research project funded and monitored by the Federal Science Policy Office of the Belgian Federal Government (Belspo). Belspo supports the Code of Ethics for Scientific Research in Belgium.

* Data for the current paper comes from four different sources from this project: (1) On-line 2012 survey; (2) On-line 2013 workshop survey; (3) Workshops 2013; (4) Secondary data (2012 and 2013) from the National Bank of Belgium. Participants gave their consent to participate in the project as follows:

(1) On-line 2012 survey

Belgian entrepreneurs received an e-mail invitation to participate in an on-line survey about ambitious entrepreneurship. They were free to participate in the survey; there was no obligation whatsoever to participate in the survey. They received the guarantee that all responses would be treated as confidential through the following statement: “*All answers will be treated as confidential!”*

By clicking on the survey link, they agreed to participate in the survey. At the end of the survey, they received the following two questions:

1. *For our research, we would like to know your company identity number (VAT number, e.g., BE0999999999). We will link your answers to publicly available company information. Only the researchers will have access to this number. The researchers will never share this number or your data with other organizations/persons. Please provide your company identity number (VAT number) below.*
2. *Have you ever wondered what the influence of personality traits and motivations is on company development? We offer you the possibility to attend a free workshop about this topic! It will be organized by outstanding research institutes, in collaboration with BELSPO, the Belgian Science Policy. Please provide the email address where we can send you more information about this seminar.*

Participants were free to fill out these questions; there was no obligation whatsoever to fill out the questions. Thus, filling out their VAT number was their own choice. By filling out this VAT number, they gave their consent to match the questionnaire data with publicly available secondary company data because we indicated the following: “*We will link your answers to publicly available company information”*.

By filling out the e-mail address to participate in a workshop about personality traits and motivations, participants gave their consent to contact them again for the workshop because we indicated the following: “*Please provide the email address where we can send you more information about this seminar”*.

(2) On-line 2013 workshop survey

Participants who indicated in the on-line 2012 survey that they wanted to participate in a workshop about personality and motivations received a follow-up e-mail. In this e-mail, we first signaled them that we got their contact details because they filled out their e-mail address in the workshop question from the on-line 2012 survey. Then, we invited them to participate in a workshop. We stated: “*A couple of months ago, you participated in a questionnaire about ambitious entrepreneurship. […] You indicated that you are interested in participating in a free workshop about entrepreneurial motives. […] You can subscribe for this workshop and choose the date and location that suits you best through the link below.”*

When subscribing for the workshop, participants were asked to fill out the explicit motives questions. We explained them that filling out these questions was necessary for us to prepare for the workshop, and stated *“To prepare for the workshop, please fill out the following questions about your personal motives. The questions refer to the need for achievement (doing something better than others), need for power (having an impact on others), and need for affiliation (being closely connected to others).”*

Participants were free to fill out these questions; there was no obligation whatsoever to fill out the questions.

(3) Workshops 2013

During the workshop, participants first received a general introduction about the project. During this introduction, we explained them again that the workshops were part of several data gathering moments; ranging from publicly available secondary (financial) company data, to on-line surveys and a series of workshops. We explained the participants that data would be linked.

After this general introduction, participants were invited to participate in a computer-led assessment. They were invited to go to the computer rooms. Participants received the information that the computer would immediately generate scores, which they could fill out on a score sheet they received from the researchers.

On this overview sheet, their individual scores on the explicit motives were already filled out. The researchers provided the following additional information on this score sheet: *“On this score sheet, you can list the scores that will be generated for you during the self-assessment tool. […] While subscribing for the workshops, you filled out an on-line survey. Your individual scores from this on-line survey are listed on this score sheet. These are your personal values.”*

By entering the computer room, the participants knew that data would be linked, and gave their verbal consent to participate in the study. Participants could stop the computer-led assessment at any time; there was no obligation whatsoever to fill out the questions.

(4) Matching with the secondary data (2012 and 2013)

As explained, participants of the on-line 2012 survey received the following two questions at the end of the survey: “*For our research, we would like to know your company identity number (VAT number, e.g., BE0999999999). We will link your answers to publicly available company information. Only the researchers will have access to this number. The researchers will never share this number or your data with other organizations/persons. Please provide your company identity number (VAT number) below.”*

Participants were free to fill out these questions; there was no obligation whatsoever to fill out the questions. Thus, filling out their VAT number was their own choice. By filling out this VAT number, they gave their consent to match the questionnaire data with publicly available secondary company data because we indicated the following: “*We will link your answers to publicly available company information”*.
