# Supplementary material for: The added value of implicit motives for management research Development and first validation of a Brief Implicit Association Test (BIAT) for the measurement of implicit motives
Source: PLoS One. 2018 Jun 20;13(6):e0198094. doi: 10.1371/journal.pone.0198094 (PMC6010206; doi:10.1371/journal.pone.0198094)
Supplement: S2 Appendix — (DOCX) [file pone.0198094.s002.docx]

**S2 Appendix. Overview of survey questions.**

Explicit motives – gathered during the on-line 2013 workshop survey

We adopted the affiliation, the dominance and the achievement sub-scales of the Personality Research Form (PRF) to assess explicit motives. The PRF is proprietary and we do not have permission to reproduce all items. Please contact “SIGMA Assessment Systems” to obtain a legal copy of the scale items. (SIGMA Assessment Systems, P.O. Box 610757, Port Huron, MI, USA 48061-0757, P.O. Box 610757, Port Huron, MI, USA 48061-0757).

Entrepreneurial self-efficacy – gathered during the 2013 workshops

On a 7-point scale from “much worse” to “much better”, how do you compare yourself to fellow entrepreneur/manager in your ability to . . .

. . . successfully identify new business opportunities?

. . . create new products?

. . . think creatively?

. . . commercialize an idea or new development?

. . . raise funds for a new business?

. . . sell a new product or service?

. . . solve other people’s problems?

. . . find new and unique ways for solving old problems?

. . . imagine different ways of thinking and doing?

. . . creating a piece of art, such as a novel, a song, a performance, or a painting?

Number of businesses and age of the entrepreneur – gathered during the on-line 2012 survey

- How many businesses did you establish (alone or together with partners)?
- What is your year of birth?
